# Practical Radiosynthesis and Preclinical Neuroimaging of [^11^C]isradipine, a Calcium Channel Antagonist

**DOI:** 10.3390/molecules20069550

**Published:** 2015-05-26

**Authors:** Benjamin H. Rotstein, Steven H. Liang, Vasily V. Belov, Eli Livni, Dylan B. Levine, Ali A. Bonab, Mikhail I. Papisov, Roy H. Perlis, Neil Vasdev

**Affiliations:** 1 Department of Radiology, Harvard Medical School, Division of Nuclear Medicine and Molecular Imaging and Center for Advanced Medical Imaging Sciences, Massachusetts General Hospital, 55 Fruit Street, Boston, MA 02114, USA; E-Mails: rotstein.benjamin@mgh.harvard.edu (B.H.R); liang.steven@mgh.harvard.edu (S.H.L.); vbelov@mgh.harvard.edu (V.V.B.); elivni@mgh.harvard.edu (E.L.); dblevine@mgh.harvard.edu (D.B.L.); bonab@pet.mgh.harvard.edu (A.A.B.); papisov@helix.mgh.harvard.edu (M.I.P.); 2 Department of Research, Shriners Hospitals for Children—Boston, 51 Blossom Street, Boston, MA 02114, USA; 3 Department of Psychiatry and Center for Experimental Drugs and Diagnostics, Massachusetts General Hospital, 185 Cambridge Street, Boston, MA 02114, USA; E-Mail: rperlis@mgh.harvard.edu

**Keywords:** carbon-11, radiosynthesis, isradipine, positron emission tomography, neuroimaging, calcium channel blocker

## Abstract

In the interest of developing *in vivo* positron emission tomography (PET) probes for neuroimaging of calcium channels, we have prepared a carbon-11 isotopologue of a dihydropyridine Ca^2+^-channel antagonist, isradipine. Desmethyl isradipine (4-(benzo[*c*][1,2,5]oxadiazol-4-yl)-5-(isopropoxycarbonyl)-2,6-dimethyl-1,4-dihydropyridine-3-carboxylic acid) was reacted with [^11^C]CH_3_I in the presence of tetrabutylammonium hydroxide in DMF in an HPLC injector loop to produce the radiotracer in a good yield (6 ± 3% uncorrected radiochemical yield) and high specific activity (143 ± 90 GBq·µmol^−1^ at end-of-synthesis). PET imaging of normal rats revealed rapid brain uptake at baseline (0.37 ± 0.08% ID/cc (percent of injected dose per cubic centimeter) at peak, 15–60 s), which was followed by fast washout. After pretreatment with isradipine (2 mg·kg^−1^, i.p.), whole brain radioactivity uptake was diminished by 25%–40%. This preliminary study confirms that [^11^C]isradipine can be synthesized routinely for research studies and is brain penetrating. Further work on Ca^2+^-channel radiotracer development is planned.

## 1. Introduction

**Figure 1:**
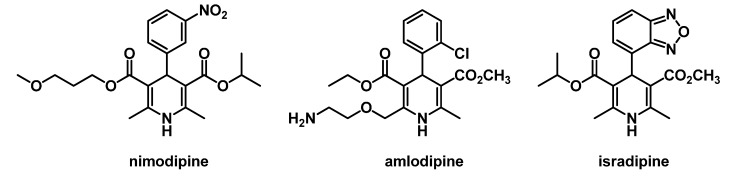
Selected 1,4-dihydropyridine (DHP) Ca^2+^-channel antagonists.

L-type calcium channels (LTCCs) are cell membrane proteins expressed in most electrically-excitable cells and involved in assorted cellular functions, including neurotransmitter and hormone secretion, Ca^2+^ homeostasis and gene expression [1,2]. A subunit of the LTCC was among the first to be associated with neuropsychiatric diseases, including schizophrenia and bipolar disorder [3,4]. While Ca^2+^-channel antagonists have a long history in the treatment of hypertension and cardiac disease [5], the promise these pharmaceuticals hold for treatment of neurological and psychiatric disorders has yet to come to fruition [6,7,8,9,10,11,12,13,14]. For example, it is believed that LTCC antagonists protect dopamine neurons in the substantia nigra from degeneration associated with Parkinson’s disease by dopamine D_2_ receptor desensitization [15]. The 1,4-dihydropyridine (DHP) scaffold has emerged as a privileged structure for LTCC blockers (Figure 1), and several of these compounds, including nimodipine, amlodipine and isradipine (PN 200-110), are among the first-line antihypertensive drugs [16]. Isradipine is currently in clinical trials for the treatment of Parkinson’s disease [17] and has demonstrated efficacy for bipolar depression in a proof-of-concept study [18].

**Figure 2:**
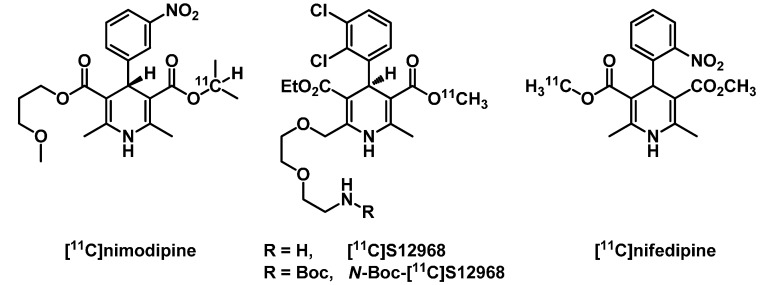
Selected DHP-based PET radiotracers.

Efforts to develop neuroimaging probes for ion channels, such as the γ-aminobutyric acid GABA_A_ receptor [19], nicotinic acetylcholine receptors (nAChRs) [20,21] and the *N*-methyl-d-aspartate (NMDA) receptor [22], have supplied a number of practical tools for clinical research and identified opportunities for subtype-selective ion channel imaging. In contrast, there remains an unmet need for an effective *in vivo* neuroimaging agent for LTCCs [23]. Several DHP-based drugs and derivatives have been radiolabeled with carbon-11 (^11^C, *t*_1/2_ = 20.4 min) or fluorine-18 (^18^F, *t*_1/2_ = 109.7 min) for positron emission tomography (PET) (Figure 2) [23,24,25,26,27,28]. Studies using these radiotracers were primarily directed toward imaging of cardiac Ca^2+^-channels, and an amlodipine-derived radiotracer, [^11^C]S12968, showed up to 80% specific binding in the myocardium [29,30,31,32,33]. [^11^C]S12968 was used for *in vivo* measurement of myocardial DHP binding site density in beagles, with low doses of Ca^2+^-channel antagonists. Unfortunately, this radiotracer does not cross the blood-brain barrier, and attempts to circumvent this limitation by manipulation of its lipophilicity were ineffective [23].

Isradipine is a highly potent DHP that serves as a reference molecule for *in vitro* studies [34] and has been shown to cross the blood-brain barrier in mice [35]. Furthermore, [*O-*^11^C-*methyl*]isradipine ([^11^C]isradipine) has previously been prepared, though this radiosynthesis demanded the production of an esoteric reagent, [^11^C]diazomethane [36]. Our goal was to evaluate [^11^C]isradipine as a potential neuroimaging agent for Ca^2+^-channels and, in the process, to develop a more convenient radiosynthesis of this molecule from commonly-used [^11^C]CH_3_I.

## 2. Results and Discussion

### 2.1. Radiosynthesis of [^11^C]Isradipine

**Figure 3:**
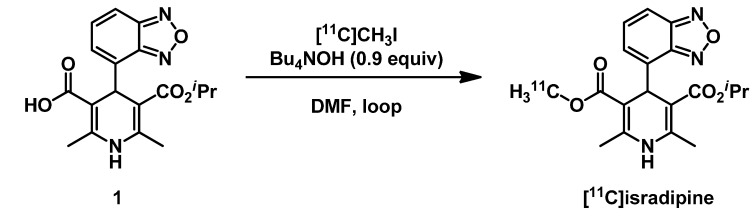
Radiosynthesis of [^11^C]isradipine.

Similar to the previous radiosynthesis, the methyl ester was identified as the most convenient site for radiolabeling isradipine with carbon-11. Using the same carboxylic acid precursor (**1**), we sought conditions for selective ^11^C-methylation using [^11^C]CH_3_I (Figure 3). Radiomethylation was conducted using the captive solvent (“loop”) method [37,38]. The HPLC injector of a commercial radiosynthesis unit was loaded with a solution of 1 mg of **1** and 0.9 equivalents of tetrabutylammonium hydroxide in 80 µL of anhydrous DMF, and [^11^C]CH_3_I was passed through the loop in a stream of helium gas. After 5 min of reaction time, the loop was flushed with the mobile phase directly onto a semi-preparative HPLC column. The radiotracer was purified and reformulated in ethanolic saline, ready for injection.

[^11^C]Isradipine was prepared in a reasonable overall yield (6 ± 3% non-decay-corrected from starting [^11^C]CO_2_) and high specific activity (143 ± 90 GBq·µmol^−1^; 3.9 ± 2.4 Ci·µmol^−1^ at end-of-synthesis). The radiosynthesis required 40 ± 2 min from end-of-bombardment and yielded the product in >95% radiochemical purity with sufficient quantities for PET imaging (1.5–10.9 GBq, 41–295 mCi). While the radiochemical purity was typically very high, we found that purified [^11^C]isradipine was susceptible to slow decomposition during the reformulation process if basic aqueous buffers were employed. Using neutral or slightly acidic buffers during reformulation, the radiochemical purity of the final product was consistently >99%. In addition to the improved accessibility of [^11^C]isradipine on account of widely available [^11^C]CH_3_I, compared with the previous report, which used [^11^C]CH_2_N_2_ [36], the radiotracer was isolated in 2–6-fold higher specific activity.

### 2.2. Partition Coefficient of [^11^C]isradipine

Octanol/buffer partition coefficients have predictive utility for assessing blood-brain barrier permeability, with an optimum log*D* range of 2.0–3.5 [39]. Owing to the presence of a basic primary amine, the log*D* of [^11^C]S12968 is 1.54, and this tracer does not enter the brain [23]. In contrast, nimodipine possesses a log*D* of 2.41 and does cross the blood-brain barrier. However, DHPs have been shown to engage in complex interactions with lipid membranes, suggesting that partition coefficients in isotropic solvent systems may hold less predictive value for this class of compounds [40]. For example, *N*-Boc-[^11^C]S12968, in which the amine is masked as a carbamate, has an increased log*D* (2.12), yet still fails to significantly enter the brain (0.04% ID/cc (percent of injected dose per cubic centimeter) at peak). With these factors in mind, we experimentally determined the log*D* of [^11^C]isradipine to be 2.15 (*n* = 8), using liquid-liquid partition between 1-octanol and phosphate-buffered saline (PBS, pH 7.4) [41].

### 2.3. Positron Emission Tomography Neuroimaging of [^11^C]Isradipine

**Figure 4:**
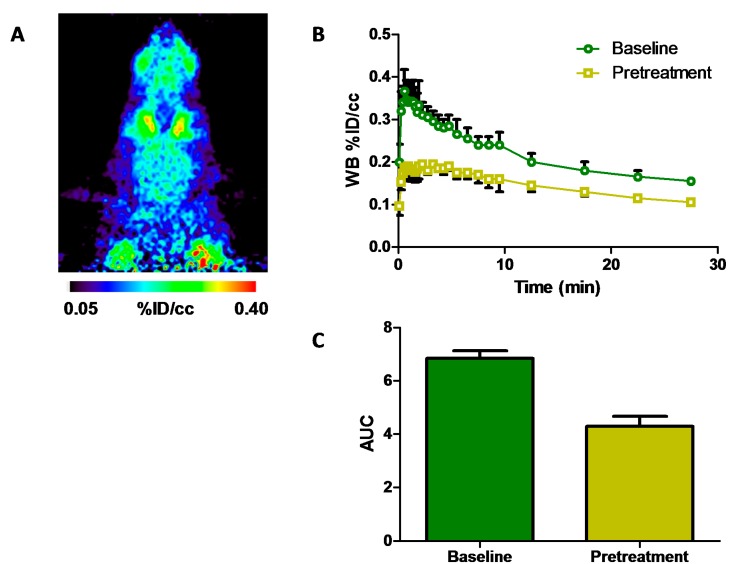
(**A**) Coronal view of summed image 0–7 min post bolus [^11^C]isradipine injection at baseline; (**B**) time-activity curves in rat whole brain at baseline (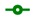) and after pretreatment (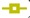) with isradipine (2 mg·kg^−1^ i.p., 30 min prior to time-of-injection (TOI)); (**C**) area-under-the-curve analysis of whole brain uptake 0–5 min after tracer injection at baseline and after pretreatment, paired *t*-test, *p* < 0.05; WB, whole brain; % ID/cc, percent of injected dose per cubic centimeter; AUC, area-under-the-curve.

To determine brain uptake and feasibility for neuroimaging of Ca^2+^-channels using [^11^C]isradipine, we conducted preliminary PET brain imaging studies in healthy, large (440–670 g), male Sprague-Dawley rats. Dynamic 30-min PET scans were acquired beginning at time-of-injection (TOI) of a bolus of radiotracer (41–166 MBq) via tail-vein, and time-activity curves were generated after image reconstruction and processing (Figure 4). Whole brain activity uptake was moderate (0.37 ± 0.08% ID/cc, percent of injected dose per cubic centimeter; 1.9 ± 0.0 SUV, standardized uptake value; *n* = 3) and peaked in the first minute after TOI. Washout was rapid, as approximately half of the peak uptake was eliminated from the brain within 20 min.

To determine the fraction of specific binding of [^11^C]isradipine *in vivo* in rat brain, animals were treated with isradipine at a dose of 2 mg·kg^−1^ i.p., 30 min prior to radiotracer administration and again imaged for 30 min. This pretreatment dose represents the upper level of previously reported i.p. dosing of isradipine [8,9], as well as the solubility threshold in vehicle (5% DMSO, 5% Tween 80, 90% saline). Whole brain uptake peaked at 0.19 ± 0.05% ID/cc (1.1 ± 0.1 SUV) 15–60 s after TOI and cleared much more slowly over the course of the imaging session. It is apparent from the time-activity curves that a significant component of whole brain uptake can be attributed to nonspecific binding. Two observations support this conclusion: (1) the magnitude of uptake after pretreatment is relatively high; and (2) the rate of clearance from 0–5 min is much slower than at baseline. The level of nonspecific binding can be estimated by area-under-the-curve (AUC) analysis. From 0–5 min post-injection of [^11^C]isradipine, uptake is approximately 60% of that at baseline (paired *t*-test, *p* < 0.05) (Figure 4C).

Given the preclinical and clinical observations of the pharmacology of isradipine and certain other DHPs, such as nimodipine, these drugs appear promising as leads for brain-penetrating Ca^2+^-channel imaging agents [8,9]. In line with previous studies, which showed that log*D* values in this range may not be strongly predictive of brain uptake for this class of compounds [23], the lipophilicities (as measured by octanol/buffer partitioning) of *N*-Boc-S12968 and isradipine are very similar, and yet, the latter shows much greater levels of brain uptake. Over the whole brain region of rats, a 40% blockade was observed after pretreatment with the nonradioactive drug. The limited effects of pre-treatment on [^11^C]isradipine uptake could be explained by numerous factors, including a low fraction of specific binding, changes in blood flow as a result of LTCC antagonism or LTCC inhibition by ethanol present in the radiotracer formulation. Further work would be required to evaluate the effects of injection vehicle, blood pressure, anesthesia and species differences on [^11^C]isradipine brain uptake, radiotracer metabolism and whether the observed specificity is uniform across all brain regions.

## 3. Experimental Section

Isradipine was purchased from U.S. Pharmacopeial Convention (USP, Rockville, MD, USA). The radiotracer precursor (4-(benzo[*c*][1,2,5]oxadiazol-4-yl)-5-(isopropoxycarbonyl)-2,6-dimethyl-1,4-dihydropyridine-3-carboxylic acid, **1**) was purchased from Aberjona Laboratories (Beverly, MA, USA). Anhydrous solvents were purchased from Fisher Scientific. Tetrabutylammonium hydroxide (1 M in methanol) was purchased from Sigma-Aldrich. A GE PETtrace 16.5 MeV cyclotron was used for [^11^C]CO_2_ production by the ^14^N(p,α)^11^C nuclear reaction using a 50-µA proton beam current to irradiate ^14^N_2_ containing 1% O_2_.

### 3.1. Radiosynthesis of [^11^C]Isradipine

Desmethyl isradipine (**1**, 1.2 mg) was dissolved in anhydrous DMF (100 µL) in a glass vial. To this vial, tetrabutylammonium hydroxide (TBAOH, 1 M MeOH, 3.0 µL, 0.9 equiv.) was added and the contents vortexed for 60 s to prepare the precursor solution. An aliquot of the precursor solution (80 µL) was withdrawn and loaded onto the HPLC injection loop of a commercial radiofluorination unit (GE Tracerlab FX N) [38]. The remaining precursor solution (20 µL) was diluted with water (20 µL), and the pH of the aqueous solution was then tested using indicator paper to confirm a pH range of 8–9. [^11^C]CH_3_I, prepared from [^11^C]CO_2_ using a commercial [^11^C]CH_3_I synthesis unit (GE Tracerlab FX MeI), was passed through the loop on a flow of He_(g)_. After transfer of [^11^C]CH_3_I was complete, gas flow was discontinued for 5 min, prior to flushing of the loop contents onto a previously equilibrated semi-preparative C18 Luna HPLC column using 6:4, CH_3_CN:0.1 M NH_4_·HCO_2_ mobile phase at 5 mL·min^−1^. The product peak was collected 10.5–11.0 min after injection into a vessel containing water (24 mL). It is noteworthy that if the bulk collection vessel contained a solution of water (22 mL) and NaHCO_3_ (8% *w*/*v*, 2 mL), a radiochemical impurity would be observed in the final product. The diluted collected fraction was passed through an HLB solid-phase extraction cartridge, which was then flushed with sterile water (10 mL). The reformulated product was collected by elution with ethanol (1 mL), followed by saline (9 mL). The product was analyzed by analytical HPLC to determine radiochemical purity and specific activity (stationary phase: C18 Luna, 5 µm, 100 Å, 250 × 4.6 mm or Prodigy ODS-3, 5 µm, 100 Å, 250 × 4.60 mm; mobile phase: 7:3, CH_3_CN:0.1 M AMF, 1 mL·min^−1^). The identity of the product was confirmed by coinjection with the known standard, isradipine.

### 3.2. PET Imaging

All animal imaging studies were performed in accordance with the National Institutes of Health Guide for the Care and Use of Laboratory Animals and were approved by the Massachusetts General Hospital Institutional Animal Care and Use Committee.

Four male rats Sprague-Dawley rats (440–670 g, Charles River Laboratories) were included in this study. Two animals were studied under both baseline and pretreatment conditions, and an additional two age- and weight-matched animals were studied under either baseline or pretreatment conditions. Animals were pair-housed on a diurnal 12:12 light/dark cycle with *ad libitum* access to food and water. Animals were weighed immediately before or immediately after imaging studies. For pretreatment studies, isradipine was reformulated in 5% DMSO, 5% Tween 80 and 90% saline and administered by intraperitoneal injection at a dose of 2 mg·kg^−1^, 30 min prior to radiotracer administration. Animals were anesthetized using isoflurane/oxygen (2%–3%, 1.5 L·min^−1^) and positioned with a custom-fabricated head holder for the duration of the imaging study (~45 min). [^11^C]Isradipine, reformulated in 0.2–1.0 mL of 10% ethanolic saline, was administered by tail-vein injection at a dose of 41–166 MBq (1.1–4.5 millicurie). Using a Siemens Focus 220 µPET (Siemens Medical Solutions, Knoxville, TN, USA) in line with a CereTom NL 3000 CT scanner (NeuroLogica, Danvers, MA, USA), list mode PET data for brain imaging were acquired for 30 min beginning at the time of radiotracer administration (TOI). Data from a second bed position, with the tail in the field-of-view of the PET camera, were then acquired in list mode for 5 min and used for correction for the activity residing in the tail. Subsequently, CT data were acquired for attenuation correction and anatomic coregistration. PET data from the 30-min scan were reconstructed using the 3D ordered-subset expectation maximization followed by maximum a posteriori reconstruction (OSEM3D/MAP) protocol (smoothing resolution of 1.5 mm, 9 OSEM3D subsets, 2 OSEM3D and 15 MAP iterations) with decay-correction to TOI and the following framing: 12 × 10 s, 6 × 30 s, 5 × 60 s, 4 × 300 s.

Reconstructed PET datasets were analyzed using AMIDE software. Elliptical regions of interest (ROI) were placed around the whole brain, as identified from overlaid CT datasets, anatomical landmarks visible in the 3D PET images and radiotracer uptake intensity. Time-activity curves were generated for each ROI and normalized to injected radiotracer dose (drawn radioactivity, less residual radioactivity in the syringe, less residual radioactivity in the tail, all decay-corrected to TOI) and expressed as the percent of the injected dose per cubic centimeter (%ID/cc) versus time. Time-activity curves were also normalized to animal weight and expressed as standardized uptake value (SUV) versus time. To facilitate comparison between subjects and treatment groups, brain radioactivity uptake was quantified at the peak (15–60 s) and evaluated by area-under-the-curve (AUC) analysis over the entire dataset and the interval showing the highest brain uptake (0–5 min).

## 4. Conclusions

Carbon-11- and fluorine-18-radiolabeled DHPs have shown utility for myocardial imaging of Ca^2+^-channels, yet imaging of this important target in the central nervous system has remained elusive. An efficient radiosynthesis of [^11^C]isradipine can be easily achieved with [^11^C]CH_3_I at room temperature using the “loop” method resulting in reasonable radiochemical yields and high specific activity. A preliminary PET neuroimaging study using this radiotracer in rats at baseline and after pretreatment with isradipine demonstrated that *in vivo* binding of this radiotracer showed only 40% blockade in whole brain. Regional analysis of [^11^C]isradipine in the brains of higher species could reveal specific binding in discrete regions of interest. The facile radiochemistry methodology developed for [^11^C]isradipine should prove to be widely applicable for *O*-11CH_3_ labeling of methyl esters of structurally-related DHPs.
